# An outbreak of leptospirosis with predominant cardiac involvement: a case series

**DOI:** 10.1186/s12879-019-3905-7

**Published:** 2019-03-18

**Authors:** P. G. N. S. Jayathilaka, A. S. V. Mendis, M. H. M. T. S. Perera, H. M. T. Damsiri, A. V. C. Gunaratne, Suneth Buddhika Agampodi

**Affiliations:** 1 North Colombo Teaching Hospital, Ragama, Sri Lanka; 2 Department of Community Medicine, Faculty of Medicine and Allied Sciences, Rajarata University of Sri Lanka, Saliyapura, Sri Lanka

**Keywords:** Leptospirosis, Myocarditis, Cardiac involvement, Co-infection

## Abstract

**Background:**

Severe leptospirosis is known to cause multi organ dysfunction including cardiac involvement. In the clinical setting with limited resources, high degree of suspicion is needed to diagnose cardiac involvement including myocarditis. Although myocarditis is not reported as a common complication due to lack of diagnostic facilities, there are evidence to support myocarditis is more prevalent in post mortem studies of patients died due to leptospirosis. We present a case series of severe leptospirosis with cardiac involvement observed during a period of one month at Colombo-North Teaching Hospital, Sri Lanka.

**Case presentation:**

We report here five patients with severe leptospirosis complicated with cardiac involvement, admitted to a single medical ward, Colombo-North Teaching Hospital, Sri Lanka during a one-month period. Out of six suspected leptospirosis patients admitted during that period, five in a raw developed severe leptospirosis with cardiac involvement. In this case series, four patients were confirmed serologically or quantitative PCR and one patient had possible leptospirosis. All patients developed shock during their course of illness. Two patients developed rapid atrial fibrillation. One patient had dynamic T wave changes in ECG and the other two had sinus tachycardia. Two patients had evidence of myocarditis in 2D echocardiogram, whereas other two patients had nonspecific findings and one patient had normal 2D echocardiogram. All five patients had elevated cardiac troponin I titre and it was normalized with the recovery. All five patients developed acute kidney injury. Four patients needed inotropic/vasopressor support to maintain mean arterial pressure and one patient recovered from shock with fluid resuscitation. All patients were recovered from their illness and repeat 2D echocardiograms after recovery did not show residual complications. One patient had serologically proven dengue co-infection with leptospirosis.

**Conclusions:**

Myocarditis and cardiac involvement in leptospirosis may be overlooked due to non-specific clinical findings and co-existing multi-organ dysfunction. Atypical presentation of this case series may be due to micro-geographic variation and unusual outbreak of leptospirosis. Co-infection of dengue with leptospirosis should be considered in managing patients especially in endemic areas.

## Background

Leptospirosis is a well-known zoonosis which causes outbreaks particularly in tropical countries. The causative organism is a spirochete of the genus Leptospira. History of leptospirosis is likely to extend to ancient times which is evident by Chinese texts describing “rice field jaundice” [1]. In 1886, Adolph Weil describes a syndrome consists of jaundice, splenomegaly, renal dysfunction, conjunctivitis, and skin rash [2] and few years later, Inada described the causative organism of *Spirochetosis icterohaemorrhegica* [3] now known as leptospirosis. The classical untreated disease is described as a biphasic illness with initial acute leptospireamic phase followed by immune phase. Most cases are self-limited, but some patients develop fatal complications with severe disease. Jaundice and renal failure (“Weil’s disease”), pulmonary hemorrhage, acute respiratory distress syndrome (ARDS), uveitis, optic neuritis, peripheral neuropathy, myocarditis, and rhabdomyolysis are well known complications [4]. After the resolution of febrile phase with the clearance of leptospiremia, the immune phase can occur in less than 10% of patients. However, atypical presentations are reported more frequently in the recent history [5]. In Sri Lanka, these differences of clinical presentations has been observed and attributed to micro-geographic changes [6]. There are more than 250 serovars of *Leptospira* which have been classified in to more than 31 serogroups and the different clinical manifestations are partially attributed to specific serovars. Understanding and identifying the varying clinical presentations of leptospirosis mimicking other diseases is important in clinical practice for early treatment and management. In this case series, we describe a series of male patients with severe leptospirosis with cardiac involvement, presented to a single medical ward during a period of one month. Here we define cardiac involvement as positivity of at least one of following criteria. They are, 1) transient echocardiogram abnormalities during the illness 2) elevated troponin I titer which came down with the recovery of illness, 3) transient electrocardiogram changes during the illness.

## Case presentation

We present five patients who were treated for leptospirosis with complications. All are male patients admitted to a single medical ward at North Colombo Teaching Hospital, Sri Lanka during a one month period starting from 31 to 01-2018. Data were collected by direct interview of patients, during admission and follow up visits, and from hospital records.

### Case 1

Fifty-eight years old previously healthy mason admitted to the hospital on 01/02/2018 with fever for three days. Fever was associated with chills, rigors, headache, body aches, faintishness, mild cough producing whitish sputum for two days, dysuria, two episodes of loose stool on day3 of illness, and loss of appetite with poor intake. Urine output was normal up to the day of admission. He had a history of cleaning a drainage system one week prior to onset of symptoms.

On examination, he was febrile (101 °F) and dehydrated. He had low volume pulse with a rate of 104 bpm, blood pressure of 84/50 mmHg. Examination of other systems were unremarkable except, few basal crepitations in the right lung.

Inward USS abdomen was performed and there was no free fluid indicative of dengue hemorrhagic fever. Initial investigations revealed neutrophil leukocytosis with thrombocytopenia (Table 1), high C-reactive protein level (Table 2), high serum creatinine with marginally elevated liver transaminases (AST > ALT). Urine analysis showed microscopic hematuria and ECG showed sinus tachycardia.

**Table 1:** Blood and urine biochemistry of five severe leptospirosis cases with cardiac manifestations

Case number	Full blood count					Renal function tests		Urine full report			Serum electrolyte (mEq/L)	
	WBC (× 10^9^/L)	PCV (%)	*N*%	Hb (g/dL)	Plt (×109/L)	S.Cr (mmol/L)	BU mmol/L	Alb	RBC/ field	PC/ field	Na	K
Case 1	7.2	40.3	87.3	14.3	103➔59	212➔ 73	10.1	1+	45–60	20–25	136	5.2➔2.2
Case 2	12.7	32	89.4	11.2	125➔81	192➔715➔247	47.9➔37.4	2+	Field full	1–2	130	6.2➔5
Case 3	12.2	38.6	91.9	13.4	125➔79	355➔114	28.9➔6.9	Trace	15–10	1–2	134	4.2
Case 4	9.13	35.4	82.5	12.5	89➔43	250➔102	20.4➔6.1	3+	10–06	30–40	140	4.1
Case 5	4.27	38.3	76.2	12.4	36➔9➔172	267➔589➔417	19.9➔18	2+	15–20	65–70	135	3.4

*WBC* white blood count, *PCV* Packed cell volume, *N* Neutrophil, *Hb* Hemoglobin, *Plt* Platelet, *S.Cr* Serum creatinine, *BU* Blood urea, *Alb* Albumin, *PC* Pus cells, *RBC* Red blood cells, *Na*- Sodium, *K* Potasium,. Arrows (➔) indicate interval change of parameters since admission to discharge

**Table 2:** Blood biochemistry of five severe leptospirosis cases with cardiac manifestations

Case number	Liver function tests							ABG		Troponin I titer (ng/mL)	CRP (mg/dL)
	AST (U/L)	ALT (U/L)	ALP (U/L)	SBR (μmol/L)	S.Alb (g/L)	INR	S.Amylase(U/L)	pH	HCO_3_^−^	Ref-0-0.12)	
Case 1	205	73	77	61	32	1.09	1574	7.46	16	3.54	248
Case 2	215	75	200	61	26	1.60	Not done	7.53	18	0.2	268
Case 3	85	48	104	31	32	1.32	Not done	7.49	13.9	6.41	360
Case 4	55	34	42	21	38	1.36	Not done	7.48	18.8	0.64	307
Case 5	131	68	87	24	30	1.25	Not done	7.48	18.5	0.124	236

*ABG* Arterial blood gas, *AST* Aspartate aminotransferase, *ALT* Alanine aminotransferase, *ALP* Alkaline phosphatase, *S.Alb* Serum albumin, *SBR* Serum bilirubin, *INR* International normalization ratio, *S.Amylase* Serum amylase, *CRP C* reactive protein

He was resuscitated with intravenous crystalloids. Despite adequate resuscitation he remained in shock and oliguric acute renal failure. After five hours of admission he was started on intravenous noradrenalin infusion and later dobutamin also added to the therapy (Table 3). Clinical diagnosis was made as leptospirosis and intravenous cefotaxime was started in the meanwhile. Urine output was improved with the rise of mean arterial pressure. But patient was dependent on ionotrope and vasopressor. 2 D echocardiogram showed mild global hypokinesia with ejection fraction 50–55% and concluded as possible myocarditis. Troponin I titre became positive. On day 4 of illness, patient developed rapid atrial fibrillation with shock requiring electrical cardioversion to achieve sinus rhythm. By day five of illness, he became heamodyanemically stable without inotropic/vasopressor support. During the recovery, he developed asymptomatic hypokalemia and potassium was replaced. By day eleven of illness he was completely recovered clinically and full blood count, liver function tests, renal function tests and ECG were normal. C-reactive protein and troponin I titer were coming down and patient was discharged. After three weeks of illness, 2D echocardiogram was performed and it was completely normal.

**Table 3:** Summary of management five severe leptospirosis cases with cardiac manifestations

Management	Fluid boluses to optimize blood pressure (fluid resuscitation)	Inotropes to maintain blood pressure		Maintenance fluid	Electrical cardio version	Medical cardio version with amiodorone	Frusemide infusion	Hemodialysis	Intravenous antibiotics	Dengue pre-critical monitoring
Case 1	Given	Noradrenaline	Dobutamine	Given	Done	Not done	Given	Not done	Cefotaxime	Done
Case 2	Given	Noradrenalin	Dobutamine	Given	Done	Given	Given	Done twice	Cefotaxime	Done
Case 3	Given	Not given		Given	Not done	Not given	Not given	Not done	Cefotaxime	Done
Case 4	Given	Noradrenalin		Given	Not done	Not given	Given	Not done	Cefotaxime	Done
Case 5	Given	Noradrenalin		Given	Not done	Not given	Given	Not done	Cefotaxime	Done

*Leptospira* was detected in qPCR (quantitative polymerase chain reaction) performed on day five of illness and leptospirosis antibody test on day seven of illness (MAT) was positive. (Titre- 1:2560) His urine and blood cultures, dengue antigen were negative.

### Case 2

A 67 years old previously healthy male, a retired clerk presented to the medical casualty with a history of fever for three days. It was associated with arthralgia, myalgia, headache and loss of appetite. He did not have respiratory, urinary symptoms and bowel habits were normal. He denied any history of exposure to leptospirosis or contact history of fever. On admission, his general examination was normal with a heart rate of 80 bpm and blood pressure of 100/70 mmHg. Other system examination was unremarkable. After admission it was noted that his urine output is low while he was on maintenance fluid. Initial investigations revealed neutrophilia with normal white blood cell count, thrombocytopenia, elevated blood urea, serum creatinine, C-reactive protein and AST. Urine analysis showed 4–6 pus cells, 1–2 red cells with granular casts. Clinical diagnosis of leptospirosis was made on high index of suspicion although there was no significant history of exposure to leptospirosis. Patient was started on intravenous cefotaxime.

By the day five of illness, he developed confusion (GCS-14/15), low blood pressure (80/40 mmHg) with tachycardia (117 bpm), high fever spike (103 F), and mild dyspnea with SpO2 98% on air. ECG showed sinus tachycardia, non-contrast CT brain was normal, 2D echocardiogram revealed ejection fraction of > 60%, chest X ray-PA was normal, and troponin I titer was marginally positive. Ultrasound abdomen showed renal parenchymal changes with normal sized kidneys. Serum creatinine was rising. Patient was started on inotropic and vasopressor support to maintain blood pressure. Even after achieving mean arterial pressure > 65 mmHg patient went in to anuric acute renal failure. Meanwhile he developed rapid atrial fibrillation which was settled with electrical cardioversion. He was given hemodialysis on day 6 of illness. On day 7 of illness again patient developed rapid atrial fibrillation and it did not respond to electrical cardioversion and started on IV amiodarone infusion and patient regained sinus rhythm and could tail off inotrope and vasopressor. Since day 8, he gradually improved clinically with good urine output, hemodynamic stability and confusion settled. But he did not recover from acute kidney injury and renal functions remained rising again. He was given another hemodialysis on day 12 of illness. Then his renal functions slowly improved and discharged on day 17 of illness with a follow up plan at nephrology clinic.

On discharge patient had normal platelet count, C-reactive protein, liver transaminases, ECG. Serum creatinine was static around 250 micromol/l. Repeat 2 D echocardiogram which was done three weeks after recovery was normal. Leptospirosis antibody titre (MAT) on day 7 of illness was positive. (1:10240).

### Case 3

A 17 year old male patient presented with fever for two days. Fever was associated with chills, rigors, arthralgia, myalgia, frontal headache, faintishness, lower back pain, loss of appetite, vomiting, loose stool 3–4 times/day for two days. Patient denied a significant exposure to leptospirosis. There was no contact history of fever. He was a manual worker. On admission he was ill looking, febrile (temp-104 F), dehydrated, mildly dyspnoec (RR-24 breaths/min) with SpO2 98% on air and had conjunctival suffusion. His pulse rate was 124 bpm with blood pressure 80/50 mmHg. Other system examination was unremarkable.

Ultrasound scan of abdomen showed acute renal parenchymal changes and there was no evidence of free fluid in the abdomen. Initial investigations revealed neutrophil leukocytosis with thrombocytopenia, high C-reactive protein (360 mg/L), high blood urea (172 mg/dL) and serum creatinine (355 micromol/L), marginally elevated liver transaminases (AST > ALT), microscopic hematuria, ECG showed sinus tachycardia with mild T inversions in V4-V6. Chest X ray was normal.

Possible diagnosis of leptospirosis was made on clinical grounds and he was started on intravenous cefotaxime. His blood pressure was improved after fluid resuscitation and he had good urine output. His 2D echocardiogram was normal, but his troponin titer increased and then came down. Patient was discharged from the ward on day 7 of illness with complete recovery and normal full blood count, renal and liver function tests. CRP and trop I titer was coming down. 2 D echocardiogram which was performed after three weeks of recovery was normal.

His dengue antigen test, blood and urine cultures were negative. The Leptospirosis qPCR test performed on day three of the illness was reported as not detected though one out of triplicate samples was positive. Patient was clinically diagnosed as a “possible” case of leptospirosis.

### Case 4

A 55 year old male laborer presented with fever for four days duration. He was previously diagnosed to have diabetes mellitus, but he was not taking treatments. Fever was associated with arthralgia, myalgia, headache, lower back pain, dysuria and reduced urine output for two days, cough for one week producing scanty amount of whitish sputum. He had a history of muddy contact within one week prior to symptom onset.

On admission, patient was febrile (temp-102F), ill looking, mildly dehydrated and had conjunctival suffusion. His pulse rate was 124 bpm with blood pressure of 90/50 mmHg. Other system examination was unremarkable. Initial laboratory work up showed neutrophilia with normal white cell count, thrombocytopenia, high C-reactive protein (250 mg/L), high serum creatinine (146 micromol/L) and normal liver transaminases. ECG showed sinus tachycardia and chest X ray-PA was normal. Depending on clinical grounds, diagnosis was made as leptospirosis and started on intravenous cefotaxime while fluid resuscitation is being carried out.

Despite adequate fluid resuscitation patient developed shock with low urine output on the same day of admission. (Day 4 of illness- Pulse rate- 130 bpm, BP-85/60) Then vasopressor support was given and small dose of frusemide infusion was started after achieving normal blood pressure with noradrenalin. 2D echocardiogram was performed on D5 of illness and it showed mild global hypokinesia with ejection fraction 45–50%, dilated left ventricle with concentric left ventricular hypertrophy and concluded as hypertensive heart disease with or without myocarditis. Cardiac troponin I titre became positive and had rising titre when repeated and then came down by the time of recovery. US scan of abdomen revealed bilateral renal parenchymal changes with normal sized kidneys. Noradrenalin was tailed off within 24 h and urine output was improved with maintenance fluid therapy. Patient had rising serum creatinine till day 6 of illness and then started to come down. Serum electrolytes were normal throughout and there was no acidosis. Patient was improved dramatically and was discharged from the hospital by day 9 of illness. On discharge he had rising platelet count, normal serum creatinine and dropping troponin I titre and CRP.

2 D echocardiogram was repeated after 4 weeks of discharge and his ejection fraction was improved to 60% and there was mild left ventricular hypertrophy with grade I diastolic dysfunction. His diabetes was controlled with soluble insulin during acute illness and changed to oral hypoglycemic treatment with the recovery. Leptospirosis antibody titre (MAT) done on day 7 of illness was positive (1:5120).

### Case 5

A 73 years old male patient presented with fever for 4 days. It was high fever associated with arthralgia, myalgia and mild difficulty in breathing. He also complained of reduced urine output and loose stool (two episodes) for one day. There were no other respiratory or urinary symptoms. He denied a significant exposure to leptospirosis. He had a past history of hypertension for which he was not taking treatment and past history of renal calculi for which he has undergone surgery several years back.

On admission he was ill looking, febrile (Temp-102 F), and anicteric. Pulse rate was 112 bpm and blood pressure 96/66 mmHg. Other system examination was unremarkable. Initial investigations revealed marked thrombocytopenia, neutrophilia with low normal white blood cell count, high C-reactive protein (236 mg/L), high serum creatinine (267 micromol/L), elevated liver transaminases (AST > ALT), urine analysis showed pus cells 65–70, red cells 15–20 and albumin 2+ (urine culture became negative). Chest X Ray-PA was normal.

Possibility of dengue fever could not be excluded with his full blood count and clinical presentation, but all other initial investigations were supportive towards leptospirosis although there was no history of significant exposure to leptospirosis. On admission ultrasound scan of the abdomen was performed inward and there was no evidence of fluid leakage. Therefore, patient was started on intravenous cefotaxime in addition to hydration with maintenance fluid. Patient had low urine output and went in to shock (PR-114, BP-78/41 mmHg) despite of adequate fluid resuscitation (on day 4 of illness). He was started on IV Noradrenalin to maintain blood pressure.

Ultrasound scan of the abdomen revealed right side scarred kidney with left side renal parenchymal changes with normal size kidney. There was no evidence of leaking by the time of developing shock. 2D echocardiogram showed severe mitral regurgitation with and there was no evidence of myocarditis. Troponin I titer became marginally positive and later came down. ECG showed sinus tachycardia. Histological diagnosis or cardiac MRI to diagnose cardiac involvement was not accessible due to lack of resources in the hospital. Noradrenalin could be tailed off within 24 h. (On day 5 of illness). By day five of illness urine output was gradually improved but serum creatinine remained rising with normal serum electrolytes. Dengue NS1 antigen was negative, but IgM and IgG antibodies were positive with dropping platelet count and white cell count (neutrophilia persisted).

Dengue pre-critical monitoring was continued while giving maintenance fluid therapy. Daily ultrasound scans were performed to exclude fluid leakage. Patient remained hemodynamically stable and platelet and white cell count started to increase by day 7 of illness and serum creatinine started to come down by day 10 of illness. He was discharged from the hospital on day 11 of illness with a plan to be followed up in nephrology clinic for possible chronic kidney disease. 2D echocardiogram was repeated after three weeks of recovery and it was normal other than trivial mitral regurgitation. Leptospirosis antibody titer done on day 7 of illness was positive. (1:2560).

## Discussion and conclusions

Severe leptospirosis is characterized by multiple organ dysfunction including liver, kidney, lungs and brain. It is also known to cause cardiac involvement as well. Cardiac manifestations range from non-specific electrocardiographic changes and arrhythmias to myocarditis, pericarditis, endocarditis and cardiogenic shock [7–9]. But the pathophysiology behind it is less well understood and the magnitude of the problem is under-reported [10].

All five patients included in this case series had evidence of acute kidney injury. The most striking feature of these five patients admitted to a single unit within a month was cardiac involvement. All five patients developed shock with low blood pressure during their course of illness. Except case number 3, all other patients needed vasopressor/inotropic support to maintain blood pressure. Case number 1 showed evidence of myocarditis in 2 D echocardiogram at the time of shock. Case number 4 had possible evidence of myocarditis whereas case number 2, 3 had normal echo findings. Case number 5 had severe mitral regurgitation in his 2D echocardiogram. All these echocardiogram were performed while the patients were in shock. Repeat 2D echocardiograms performed after three weeks of recovery were completely normal except in case number 4 and 5. Number 4 had mild left ventricular hypertrophy with grade 1 diastolic dysfunction and number 5 had trivial mitral regurgitation. In addition to these various echo findings all of these five patients had more or less positive cardiac troponin I titre which came down with the recovery of illness. Case number one and two developed atrial fibrillation which needed intervention for normalization. Case number three had mild T wave inversions in anterior leads which was dynamic in serial electrocardiograms. Case number 4 and 5 had only sinus tachycardia. All five patients had shock by definition and the most probable explanation is cardiogenic shock due to cardiac involvement of leptospirosis. Though not commonly reported, myocarditis in severe leptospirosis may not be a rare complication.

The European Society of Cardiology working group on myocardial and pericardial diseases has developed clinical and diagnostic criteria, when present myocarditis should be suspected. Presence of unexplained cardiogenic shock, positive cardiac troponins, variable ECG changes are included for these criteria in addition to several other criteria [10]. Definitive diagnosis of myocarditis ideally should be established by histopathological, immunological and immunohistochemical criteria for which myocardial biopsy is required. This is not practical in most settings as these investigations are not routinely done and not required for patient management. In this case series none of the patients underwent histopathological or cardiac MRI diagnosis of cardiac involvement due to lack of resources in the hospital. Due to wide variability in presentation and non-specific clinical findings, many cases of myocarditis likely to go undetected. As an example, study conducted examining 24 hearts from patients who had died due to leptospirosis has revealed myocarditis in 96% of cases histologically. Endocardial inflammation had been observed in 50% of cases [11]. In Sri Lanka, myocarditis has been reported previously as a complication of leptospirosis [12, 13] and around 7–15% of confirmed cases are being reported as having this complication [6, 14, 15]. However, in most of the previous studies, the details of diagnosis of myocarditis was not clearly given. In our case series, histological diagnosis or cardiac MRI to diagnose cardiac involvement was not possible due to lack of resources in the hospital.

There is another phenomena coming up in the recent literature to explain the shock in leptospirosis. According to Julie Cagliero et al. dys-regulation of inflammatory mechanisms in severe leptospirosis can lead to cytokine storm causing sepsis like picture [16]. Systemic inflammatory response syndrome (SIRS) is supposed to occur in severe leptospirosis [17]. SIRS itself can cause elevated cardiac troponins [18, 19]. Therefore, pure cardiac involvement in leptospirosis becomes more difficult to diagnose.

All these five patients presented during one-month period in a raw and we had only six total suspected (notified) cases of leptospirosis during that month. Observing cardiac involvement in five out of six probable cases of leptospirosis may be due to an outbreak caused by a different strain of a *Leptospira*. As previously observed, outbreaks of leptospirosis with uncommon complications such as pancreatitis [5] needs more investigations and explanations. However these patients did not have evidence of pulmonary involvement which is a known complication to occur in severe leptospirosis.

Case number 5 patient had serological evidence of leptospirosis and co-infection with dengue virus. Co-infection of leptospirosis and dengue is a known phenomenon in endemic countries with subtropical and tropical climates. A study conducted in Malaysia has concluded that there is a considerable prevalence of leptospirosis and dengue co-infection with overlapping demographic, clinical and laboratory presentations [16]. In Sri Lanka [17] as well as in many other places [18–20], a co-infection of these two had been reported earlier and possible due to high endemicity of both diseases. It is crucial to consider co-infection with dengue where clinical suspicion arise even in the presence of enough supportive evidence for leptospirosis. Because close monitoring and fluid management are the lifesaving principles of management of dengue hemorrhagic fever which must be done timely.

Developing severe leptospirosis in five out of six cases during same period may be due to outbreak of uncommon strain of leptospirosis. Cardiac manifestations of leptospirosis are possibly under-diagnosed due to co-existence with other multi-organ involvement. Diagnosis of myocarditis is difficult due to lack of imaging facilities, lack of specificity of available tests as well as unavailability of non-invasive gold standard diagnostic test. To assess the significance of cardiac troponins in diagnosing cardiac involvement in leptospirosis further studies are required. Co-infection of dengue in a patient with leptospirosis should be considered especially in endemic areas.

## Abbreviations

ALT: Alanine aminotransferase
AST: Aspartate aminotransferase
BP: Blood pressure
CRP: C-Reactive protein
CT: Computed tomography
ECG: Electrocardiogram
GCS: Glasgow coma scale
IV: Intravenous
MAT: Microscopic agglutination test
MRI: Magnetic resonance imaging
qPCR: quantitative polymerase chain reaction
RR: Respiratory rate
USS: Ultrasound scan
